# Patient-Reported Outcomes in Online Communications on Statins, Memory, and Cognition: Qualitative Analysis Using Online Communities

**DOI:** 10.2196/14809

**Published:** 2019-11-28

**Authors:** Farris Timimi, Sara Ray, Erik Jones, Lee Aase, Kathleen Hoffman

**Affiliations:** 1 Department of Cardiovascular Disease Mayo Clinic Rochester, MN United States; 2 Inspire Arlington, VA United States

**Keywords:** social media, hydroxymethylglutaryl-CoA reductase inhibitors, drug-related side effects and adverse reactions, memory loss, PROMs, pharmacovigilance, infodemiology, infoveillance, peer-support groups

## Abstract

**Background:**

In drug development clinical trials, there is a need for balance between restricting variables by setting eligibility criteria and representing the broader patient population that may use a product once it is approved. Similarly, although recent policy initiatives focusing on the inclusion of historically underrepresented groups are being implemented, barriers still remain. These limitations of clinical trials may mask potential product benefits and side effects. To bridge these gaps, online communication in health communities may serve as an additional population signal for drug side effects.

**Objective:**

The aim of this study was to employ a nontraditional dataset to identify drug side-effect signals. The study was designed to apply both natural language processing (NLP) technology and hands-on linguistic analysis to a set of online posts from known statin users to (1) identify any underlying crossover between the use of statins and impairment of memory or cognition and (2) obtain patient lexicon in their descriptions of experiences with statin medications and memory changes.

**Methods:**

Researchers utilized user-generated content on Inspire, looking at over 11 million posts across Inspire. Posts were written by patients and caregivers belonging to a variety of communities on Inspire. After identifying these posts, researchers used NLP and hands-on linguistic analysis to draw and expand upon correlations among statin use, memory, and cognition.

**Results:**

NLP analysis of posts identified statistical correlations between statin users and the discussion of memory impairment, which were not observed in control groups. NLP found that, out of all members on Inspire, 3.1% had posted about memory or cognition. In a control group of those who had posted about TNF inhibitors, 6.2% had also posted about memory and cognition. In comparison, of all those who had posted about a statin medication, 22.6% (*P*<.001) also posted about memory and cognition. Furthermore, linguistic analysis of a sample of posts provided themes and context to these statistical findings. By looking at posts from statin users about memory, four key themes were found and described in detail in the data: memory loss, aphasia, cognitive impairment, and emotional change.

**Conclusions:**

Correlations from this study point to a need for further research on the impact of statins on memory and cognition. Furthermore, when using nontraditional datasets, such as online communities, NLP and linguistic methodologies broaden the population for identifying side-effect signals. For side effects such as those on memory and cognition, where self-reporting may be unreliable, these methods can provide another avenue to inform patients, providers, and the Food and Drug Administration.

## Introduction

### Background

Upon the implementation of the American College of Cardiology–American Heart Association’s guidelines published in 2013, it was estimated that 1 billion people worldwide would be eligible to take statins to prevent cardiovascular diseases [1,2]. In fact, hydroxymethylglutaryl–coenzyme A reductase inhibitors, or statin medications, have become one of the most commonly prescribed classes of medications in the United States. Often positioned as safe and effective, their prevalence and relative acceptance do not mean that patients are without risk. Commonly cited statin-associated symptoms include muscular complaints (pain, cramps, and muscle weakness) [3-5], diabetes mellitus [6,7], and changes in memory or cognition [8-12].

Examples of research investigating the effects of statins on the central nervous system include case studies [8], observational studies [9], and randomized clinical trials [10-12] with meta-analyses reporting contradictory results [13,14]. Unfortunately, a discrepancy lies between the reported degree of memory changes and the actual observed frequency in large-scale clinical trials.

However, in 2012, on the basis of a review of spontaneous reports of amnesia, confusion, and concentration complaints, among others, from the Food and Drug Administration’s (FDA) Adverse Event Reporting System, the FDA required a statin label change [15]:

Memory loss and confusion have been reported with statin use. These reported events were generally not serious and went away once the drug was no longer being taken.

Many feel the evidence is inconclusive and the decision remains controversial.

In drug development clinical trials, there is a need for balance between restricting variables by setting eligibility criteria and representing the broader patient population that may use a product once it is approved. Similarly, although recent policy initiatives focusing on the inclusion of historically underrepresented groups are being implemented, barriers still remain. These limitations of clinical trials may mask potential product benefits and side effects. To bridge these gaps, online communication in health communities may serve as an additional population signal for drug side effects.

Using these novel data sources requires unique strategies. There is a wealth of patient experience data to be found in online patient forums. The data here are unstructured, allowing for naturally occurring themes and topics to be identified through organic patient and caregiver language.

Natural language processing (NLP) as well as hands-on linguistic analysis can be applied to online posts. In this study, posts were authored by patients and caregivers on Inspire, a company that creates and manages online support communities for more than 1 million patients and caregivers. Analysis of online patient and caregiver communications discussing statins utilized NLP methodology and technology to draw correlations among discussions of memory events by statin users and compared these events with discussions of memory events of patients using other classes of medication as well as discussions of memory events of all other patients on Inspire. The NLP system uses tokenization, lemmatization, stemming, edit distances, acronym dissection, and word and phrase boundaries to understand the content and meaning of the posts. These findings were then associated with Wikipedia entries and correlated with a dictionary of conditions and treatments from the National Institutes of Health (developed in partnership with Stanford University) to accurately extract the entities used in this study. In addition, manual curation of posts using linguistic analysis provided qualitative context to these statistical findings.

By applying these tools to posts created by members on Inspire who belonged to communities focused on heart health, the data could be used to look at statin medications and identify any underlying crossover between the use of statins and impairment of memory or cognition. Moreover, this combined strategy accessed the patient’s voice, providing a detailed guide to how community members describe their experiences with statin medications and memory changes.

### Objective

The objective of this study was to employ a nontraditional dataset to identify drug side-effect signals. Specifically, the study was designed to apply both NLP technology and hands-on linguistic analysis to a set of online posts from known statin users (1) to identify any underlying crossover between the use of statins and impairment of memory or cognition and (2) to obtain patient lexicon in their descriptions of experiences with statin medications and memory changes.** **

## Methods

Researchers utilized user-generated content (UGC) on Inspire, looking at over 11 million posts across it. Posts were written by patients and caregivers belonging to a variety of communities on Inspire. After identifying these posts, researchers used NLP and hands-on linguistic analysis to draw correlations between statin use and memory and cognition.

### Natural Language Processing and Statistical Comparison

**Figure 1:**
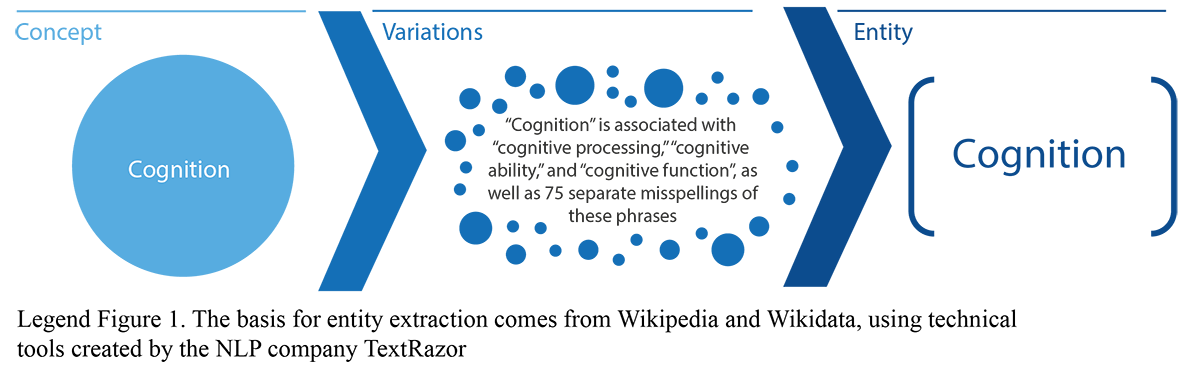
Creation of entities. The basis for entity extraction comes from Wikipedia and Wikidata, using technical tools created by the natural language processing company TextRazor.

In the corpus of over 11 million unique posts and over 440,000 different posters (patients and caregivers—*authors* of posts) on Inspire, the researchers used an NLP system that extracted relevant entities from each post. Entities are words or phrases that relate to each concept under consideration. For example, if the concept under consideration is cognition, several words or phrases related to that concept result in the creation of an entity (see Figure 1).

The first stage of the analysis was to extract every post that contained an entity that related to memory loss or cognitive decline. The following are the entities that were used, each one of which has a distinct entry on Wikipedia: cognition, cognitive disorder, memory, recall, short-term memory, memory disorder, working memory, memory span, severe cognitive impairment, mild cognitive impairment, and cognitive deficit.

Note that each entity is associated with dozens of variations, that is, different phrasings, colloquialisms, and misspellings. For instance, *cognition* is associated with *cognitive processing*, *cognitive ability*, and *cognitive function*, as well as 75 separate misspellings of these phrases.

The researchers then did the same with statins, using the following entities: ulinastatin, cilastatin, niacin, simvastatin, fluvastatin, migrastatin, follistatin, mevastatin, oncostatin M, cystatin, cerivastatin, somatostatin, simvastatin, lovastatin, combretastatin A4 phosphate, myostatin, nystatin, atorvastatin, amlodipine, angiostatin, Crestor, Lipitor, and Vytorin.

Finally, the team identified medication discussions that could serve as the control group. The researchers wanted to choose a baseline of posts that indicated people were writing about a specific medication to reduce the experimental variation to a single change, specifically the medication being discussed. In this case, the decision was made to use Tumor Necrosis Factor TNF inhibitors.

TNF inhibitors were chosen for this statistical comparison for several reasons. First, TNF inhibitors are a commonly prescribed medication, used by a significant portion of Inspire members. Second, TNF inhibitors are prescribed for conditions that do not overlap with the conditions that statins treat; therefore, there would be as little overlap as possible between people who take both types of medications. Third, TNF inhibitors have shown no association with memory loss or cognitive decline. Fourth, generally the same age cohort uses TNF inhibitors and statins, minimizing age-related effects of cognitive decline between the 2 groups.

The entities used for TNF inhibitors were the following: Humira, golimumab, Enbrel, certolizumab pegol, and Remicade.

By finding authors who had written about one or more entities from each set and authors who had written posts that contained overlap among entities in multiple sets, the researchers were able to perform significant statistical analysis among the sets. It was irrelevant to the analysis as to what was mentioned first. An author could mention cognitive issues at one point in time and then later mention statins, or it could happen in the reverse order.

### Hands-On Linguistic Analysis

To provide qualitative context to statistical findings, manual curation using linguistic methodology was performed on a subset of 246 UGC posts that mentioned both statin use and memory events. Posts were extracted and placed within an analyzable Excel file containing the following information: anonymized user identifier, date of post, title of the post, link to the post, and content of the post. Researchers developed a data-driven codebook with code label, full definition, and an example. Following the process described by Boyatzi, the team first reviewed and reduced the raw information; second, identified subsample themes; third, compared themes; fourth, created codes; and fifth, determined the reliability of the codes [16]. This comprised tags around top topics, challenges, and descriptors to identify patient themes around the type of memory impairment, experience of impairment, and the patient lexicon. Overall, 2 separate researchers applied the codebook to the data. Calculating reliability as the number of agreements divided by the total number of agreements plus disagreements resulted in intercoder reliability of 90%.

### Ethics Statement

The study was reviewed internally by Mayo Clinic and found to be exempt from the review the of Institutional Review Board. All personally identifiable information was removed. All data were evaluated without the knowledge of the identity of those involved.

## Results

NLP analysis of posts identified statistical correlations between statin users and discussion of memory impairment, which were not seen in control groups. Furthermore, linguistic analysis of a sample of posts provided themes and context to these statistical findings.

### Natural Language Processing and Statistical Comparison

Through the analysis described above, the researchers found the following results for the number of people who had posted about one of these topics on Inspire (see Table 1).

To test the statistical significance of the observed high proportion of members that had a post that included the statins entity and that had a post that included memory, Fisher exact test (calculated in R, version 3.4.1) was applied. The calculated odds ratio was 9.703 (*P* value <.001), with a 95% CI of 9.066 to 10.378. This is outlined in a 2-way contingency table (Table 2) used to calculate the odds ratio and the associated significance measures.

**Table 1:** Inspire member numbers by post topic.

Inspire member numbers	Posts about anything	Posts about Tumor Necrosis Factor inhibitors	Posts about statin medication
Total members, n	440,835	14,323	5259
Subset of members who posted about memory, n (%)	13,878 (3.15)^a^	884 (6.17)^a^	1186 (22.55)^a^

^a^Percentage of overlap.

**Table 2:** Two-way contingency table calculating odds ratio and significance measures.

Entities	Memory entity	No memory entity	Sum
Statins entity	1186	4073	5259
No statin entity	12,692	422,884	435,576
Sum	13,878	426,957	440,835

 

### Hands-On Linguistic Analysis

A total of 4 key themes related to the authors’ memory were found in the data: memory loss, aphasia, cognitive impairment, and emotional change. Summaries of these themes, patient lexicon, and specific examples can be found in Table 3. Within these themes, descriptions of memory loss and confusion often overlap, and authors may label moments of cognitive impairment, confusion, or difficulties with function as memory loss. Regardless of the labels, authors were passionate about memory and cognitive impairment. Many used qualifiers of severity and speed, such as *catastrophic* or *instant*, when describing effects on their memory. Furthermore, a subset described their experience as *dementia* or *Alzheimer’s*.

Memory difficulties were also attributed to aging, *pumphead* (postperfusion syndrome), surgery, or other medications. Authors who did attribute statin use to memory loss believed higher doses to be riskier. Authors also believed that their cognitive changes would resolve when statin use was discontinued. Nevertheless, during use, many memory events may have been written off by patients or may have gone unreported. Compounding the problem, authors reported having difficulty discussing their memory or cognitive issues with the doctors. Authors reported that many of their health care providers were dismissive of their claims, feeling that the benefits of statin medications far outweigh the risks.

**Table 3:** Patient lexicon and examples of key memory themes.

Key memory theme	Patient lexicon	Example
Memory loss: Though some patients have trouble with long-term memory, these patients are most passionate about and focused on the impact on short-term memory.	Short-term/long-term memory loss Reduced short-term memory Short-term memory comes and goes Memory problems Memory issues Memory impaired Memory difficulties Memory was shot Decline in memory Forgetful Trouble remembering things	Forgetting things that occurred, had been done, or were supposed to be done in the future Forgetting things/facts authors are supposed to know
Aphasia: Authors describe difficulty communicating their thoughts. In particular, authors have difficulty remembering names of people that they recognize and know well.	Verbal thinking Loss of words Word finding or unable to find words	Difficulty recalling words, particularly names Difficulty forming sentences
Cognitive impairment: Authors describe a loss of ability to think, reason, or understand. Authors are also concerned with the loss of ability to function. Authors are particularly concerned about attention span and forgetting how to do basic tasks.	Confusion/mental confusion Alzheimer’s/instant Alzheimer’s Dementia Brain fog/foggy Cognitive loss Neurological issues Fuzzy thinking Slow Cognitive problems Senile Mental focus Cognitive impairment	Loss of focus or attention span Inability to type or write Difficulty counting money or shopping Tasks take longer to accomplish or forgetting how to do a task Getting into the wrong car or being unable to identify one’s car Wandering around in the night and not knowing why Inability to recognize people Cannot recognize known people Difficulty explaining things to others, particularly doctors
Emotional change: Authors describe their emotions as changed, struggling with feeling depressed or lack of emotion. Authors also describe becoming angry easily or being moody or disinterested. A small subset feels increased anxiety.	Depression/sad Quick temper Moody/moodiness Anxious Tired Emotional lability	Loss of desire or trouble feeling happy Overreacting to situations Becoming angry quickly Worry Feelings of ambivalence

## Discussion

### Principal Findings

This study uses online communities of self-identified patients and caregivers to evaluate the signals of self-reported memory impairment in statin users. A cohort of statin users was compared with a cohort of patients using TNF inhibitors and also to the patients on the site overall. Though discussions of memory impairment occurred 3.1% and 6.2% of the time in the overall population and the TNF inhibitor users, respectively, overlap between the statin user conversation and memory impairment posts was 22.6%, indicating a much higher and significantly different correlation between those on statin medications and the discussion of memory issues.

Furthermore, linguistic analysis was performed on a set of posts from statin users that discussed memory impairment to identify key themes. These patients and caregivers identified difficulty with memory loss, aphasia, cognitive function, and emotional change. Patients and caregivers indicated the speed and severity of these changes, likening their experience to *instant Alzheimer’s*.

Although there are clinical trials that have not found associations of cognitive impairment with statin use, those trials were specifically conducted to evaluate cardiovascular outcomes, not cognitive outcomes [17-19]. During the postmarketing phase, a double-blind study comparing statins with placebo found small but significant differences in neuropsychological tests on attention and psychomotor speed [11]. Using the Naranjo adverse drug reaction (ADR) probability scale in a survey of 171 patients on statins, 75% of the participants were found to have experienced cognitive ADRs [20]. Another analysis involved examination of the FDA’s Adverse Event Reporting System. It revealed a significantly higher proportion of adverse reports for statins as compared with control medications [21].

This study’s findings support the importance of using novel and nontraditional data sources as additional population signals for side effects. The nature of memory issues and cognitive impairment can lead to underreporting of these issues to doctors. Furthermore, in the online posts, patients report doctors discounting, ignoring, or not taking their concerns over memory issues seriously. Improved patient care and outcomes are seriously impaired when their concerns are discounted. Underreporting of this side effect to the FDA is inevitable when patient complaints and concerns are not addressed. This underscores the imperative of bringing the patient experience to light in nontraditional ways.

Drug development clinical trials should balance eligibility criteria, which allow for a defined population to be studied, against the limitations these criteria create. Specifically, eligibility criteria exclude and narrow, reducing the representativeness of the data. This subset of population may not represent the broader patient population that may use a product once it is approved. Although there have been recent policy initiatives that focus on the inclusion of historically underrepresented groups, barriers still remain that may limit or mask the potential benefits and side effects, respectively. Data provided from online communities may allow for an expansion of cohorts for study, potentially accessing patients who may not have traditionally been enrolled in other forms of research. Analyzing data from these patients and using mixed quantitative and qualitative methodologies allows corroboration of findings and provides a new avenue of side effect identification and investigation.

### Limitations

This study had some limitations. First, clinical diagnosis, medical history, and current treatment of individual authors are self-reported, cannot be confirmed, and may be missing some information. Second, demographic data of the authors are unknown, making it unclear how the data reflect the general population. In addition, there may be an element of detection bias that may be relevant, given the intrinsic virality of online communication. Moreover, qualitative analysis may also reflect a limitation of outcome misclassification predicated upon word selection on the part of participants. Search terms were selected based on free-text review to limit the impact of alternative term selection. Despite this, missed cases may be a problem if community participants chose not to discuss or share memory challenges owing to perceived associated stigma.

One strength of qualitative research, detailed information about the human experience, makes it a compelling tool when applied to health [22]. The large datasets of online communication on social media sites, such as Inspire, can challenge the skills of individual researchers [23]. Maintenance of rigor in analysis may be impacted. Using NLP can effectively deal with this limitation.

However, in a methodological research study comparing NLP-only analysis, qualitative text–only analysis, and combined NLP–qualitative text analysis, researchers concluded that NLP-only analysis was an effective tool to identify and quantify major themes but lacked the ability to capture contextual nuances necessary for clarity and understanding. Combining the 2 methodologies provided the most comprehensive and highest quality results [24].

### Comparison With Prior Work

Stanford University Medical School in collaboration with Inspire conducted research on over 8 million posts on Inspire utilizing NLP to look for associations between chemotherapeutic agents and ADRs. The research specifically extracted mentions of common and rare cutaneous ADRs (eg, rashes, blisters, and psoriasis flares) from posts related to (1) the epidermal growth factor receptor inhibitor, erlotinib, and (2) the immune checkpoint programmed cell death–1 inhibitors, nivolumab and pembrolizumab. The team discovered that some patients receiving the chemotherapy drug erlotinib (Tarceva) reported hypohidrosis—the inability to sweat—a condition that can lead to heat exhaustion, heat stroke, or even death. This ADR had never been reported in the medical literature, but Inspire members had been discussing it for over 11 years. The team also found that Inspire members discussed, among themselves, ADRs for other checkpoint inhibitors much earlier than reported in the medical literature—an average of 7 months before any of these side effects had been reported [25]. This research demonstrated the untapped resources and information to be uncovered in online health community postings.

### Conclusions

Estimates of cessation of statin therapy within the first year are as high as 50%. This high degree of discontinuation is disconcerting as statins have been shown to reduce cardiovascular disease risk, accruing with each year of use and benefits persisting over the long term [26]. Side effects play a role in medication cessation. Although the benefits of statin medications may outweigh the risks, it is still worth identifying adverse reactions to better and more fully inform patient and doctor decisions.

Using the deidentified communication occurring online can add to the knowledge base about medication usage and adverse reactions. Large online populations, comprising large cohorts of patients who may not traditionally participate in research, can serve as additional population signals for side effects.

As with the prior work with erlotinib, this type of research can be used to inform the FDA of the side effects or adverse reactions that are presently unreported for a number of reasons. Using the combination of NLP analysis with qualitative analysis broadens and deepens learnings, tests hypotheses, and uncovers insights into the patient experience. Regarding statin use, patients may not connect their medications to cognitive changes, may not feel comfortable informing their physicians, or may be unable to articulate these changes in the clinical setting. If patients do tell the doctors, doctors may not relay this information to the FDA or feel that benefits outweigh the risks.

Further research is needed to truly identify the extent of cognitive change that is occurring with the use of statins.

## Abbreviations

ADR: adverse drug reaction
FDA: Food and Drug Administration
NLP: natural language processing
TNF: Tumor Necrosis Factor
UGC: user-generated content
